# The genome sequence of the lesser white-fronted goose, *Anser erythropus* (Linnaeus, 1758) (Anseriformes: Anatidae)

**DOI:** 10.12688/wellcomeopenres.26718.1

**Published:** 2026-06-13

**Authors:** Sinan Julian Keleş, Michelle F. O’Brien, Rosa Lopez Colom, Natural History Museum Genome Acquisition Lab, Wellcome Sanger Institute Tree of Life Management, Samples and Laboratory team, Wellcome Sanger Institute Scientific Operations: Sequencing Operations, Wellcome Sanger Institute Tree of Life Core Informatics team, Tree of Life Core Informatics collective, Darwin Tree of Life Consortium

**Affiliations:** 1 Wildfowl and Wetlands Trust, Slimbridge, England, UK; 2 Twycross Zoo, Atherstone, Warwickshire, England, UK

**Keywords:** Anser erythropus, lesser white-fronted goose, genome sequence, chromosomal, Anseriformes

## Abstract

We present a genome assembly from an individual female *Anser erythropus* (lesser white-fronted goose; Chordata; Aves; Anseriformes; Anatidae). The assembly contains two haplotypes with total lengths of 1 339.88 megabases and 1 168.02 megabases. Most of haplotype 1 (88.67%) is scaffolded into 41 chromosomal pseudomolecules, including the W and Z sex chromosomes. Haplotype 2 was assembled to scaffold level. The mitochondrial genome has also been assembled, with a length of 16.74 kilobases. This assembly was generated as part of the Darwin Tree of Life project, which produces genomes for eukaryotic species found in Britain and Ireland.

## Species taxonomy

Eukaryota; Metazoa; Chordata; Aves; Anseriformes; Anatidae; *Anser*; *Anser erythropus* (Linnaeus, 1758) (NCBI:txid132586).

## Background

The lesser white-fronted goose ( *Anser erythropus*), the smallest of the “grey” geese, is an important wetland indicator species and a long-distance migratory waterbird ( Kear, 2005; Liu *et al.*, 2023). It has a pink bill with a white facial blaze at its base, black belly patches, and a yellow eye-ring ( BirdLife International, 2018). Body length ranges from 53–66 cm, and males and females are similar in appearance (sexual monomorphism), though males tend to be slightly larger, with wing lengths averaging 378 mm compared to 373 mm in females, and weights ranging from 1 950–2 300 g and 1 400–2 150 g respectively ( Madge & Burn, 1988). At first glance, lesser white-fronted geese can be mistaken for greater white-fronted geese ( *Anser albifrons*), which are generally larger, with longer necks, bigger bills, and flatter foreheads and lack the bright yellow eye-ring characteristic of *A. erythropus* ( BirdLife International, 2018).

Lesser white-fronted geese inhabit a variety of landscapes, including inland shrub-, grass- and wetlands, rocky areas such as cliffs and mountain peaks, and agricultural pastures ( BirdLife International, 2021). They breed in bogs, tundra, taiga edges and alpine foothills near wetlands (≤700 m above sea level), nesting on early snow-free ground close to open water ( BirdLife International, 2021). Nests are shallow ground scrapes lined with down and vegetation, often reused across seasons, and typically contain four to six eggs ( BirdLife International, 2021; Kear, 2005). Their diet is mainly herbivorous, consisting of terrestrial and aquatic plants, and is supplemented with agricultural grains during winter ( BirdLife International, 2021; Kear, 2005). Main food sources vary geographically, including grasses of the family Poaceae in the grasslands of Shengjin Lake, and *Carex* species and subterranean tubers in the mudflats of Caizi Lake ( Zhao *et al.*, 2013). The species has a generational length of approximately 11.4 years ( BirdLife International, 2018).

In 2018, the global population of *Anser erythropus* was estimated at 16 000 to 27 000 individuals ( BirdLife International, 2018), although estimates vary. Tian *et al*. (2021) describe three recognised populations, corresponding to distinct migratory flyways: The Fennoscandian population, spanning Norway to the Kola Peninsula, is very small, with only 40 to 50 pairs, including a reintroduced population in Sweden ( Andersson & Holmqvist, 2010; Fox & Leafloor, 2018; Marolla *et al.*, 2019). The main western population, ranging from northwest Russia to Taimyr, is much larger, numbering 30 000 to 34 000 individuals ( Cuthbert *et al.*, 2018; Fox *et al.*, 2010; Rozenfeld *et al.*, 2019). The eastern population, from east of Taimyr to Chukotka, is estimated at 14 000 to 19 000 and is currently in decline ( BirdLife International, 2018; Fox & Leafloor, 2018; Jia *et al.*, 2016). It is noteworthy that the Fennoscandian and western (Russian) populations are genetically indistinguishable ( Díez-Del-Molino *et al.*, 2020). During summer, *A. erythropus* breeds in regions from northern Scandinavia to northeastern Siberia and migrates to wintering areas in southeast Europe, the Middle East, Japan, China, and South Korea ( BirdLife International, 2021; Liu *et al.*, 2023).

*Anser erythropus* is listed as “vulnerable” on the IUCN Red List, with a declining population trend ( BirdLife International, 2021). Zhao *et al.*, (2013) note that *A. erythropus* is considered a lake ecosystem indicator species, due to its sensitivity to habitat change. Threats to this species are multifactorial, including human disturbances such as agriculture and aquaculture, hunting, recreational activities, habitat loss and degradation, and ecosystem disturbances (e.g. dams or other water-level modifications), as well as pollution. Natural predation by red foxes and large raptors also contributes to population pressures ( BirdLife International, 2021; Jones *et al.*, 2008; Wang *et al.*, 2012; Zhang *et al.*, 2020; Zöckler & Lysenko, 2000).

Following the discontinuation of a Swedish *Anser erythropus ex-situ* breeding programme ( von Essen, 1991) – prompted by the detection of greater white-fronted goose ( *A. albifrons*) mitochondrial DNA within the breeding stock ( Ruokonen *et al.*, 2000) – Díez-Del-Molino *et al.* (2020) conducted a population genomics study. Their analyses revealed no detectable introgression of *A. albifrons* genes into the Swedish population of lesser white-fronted geese, highlighting the importance of genetic investigations. Such work provides scientific evidence that can guide conservation programme planning and improve management outcomes.

We present a chromosome-level genome sequence for *Anser erythropus.* Another scaffold-level assembly for this species is also available (GCA_051252735.1), submitted by Iridian Genomes (NCBI datasets, O’Leary *et al.*, 2024).

## Methods

### Sample acquisition

**Figure 1.**
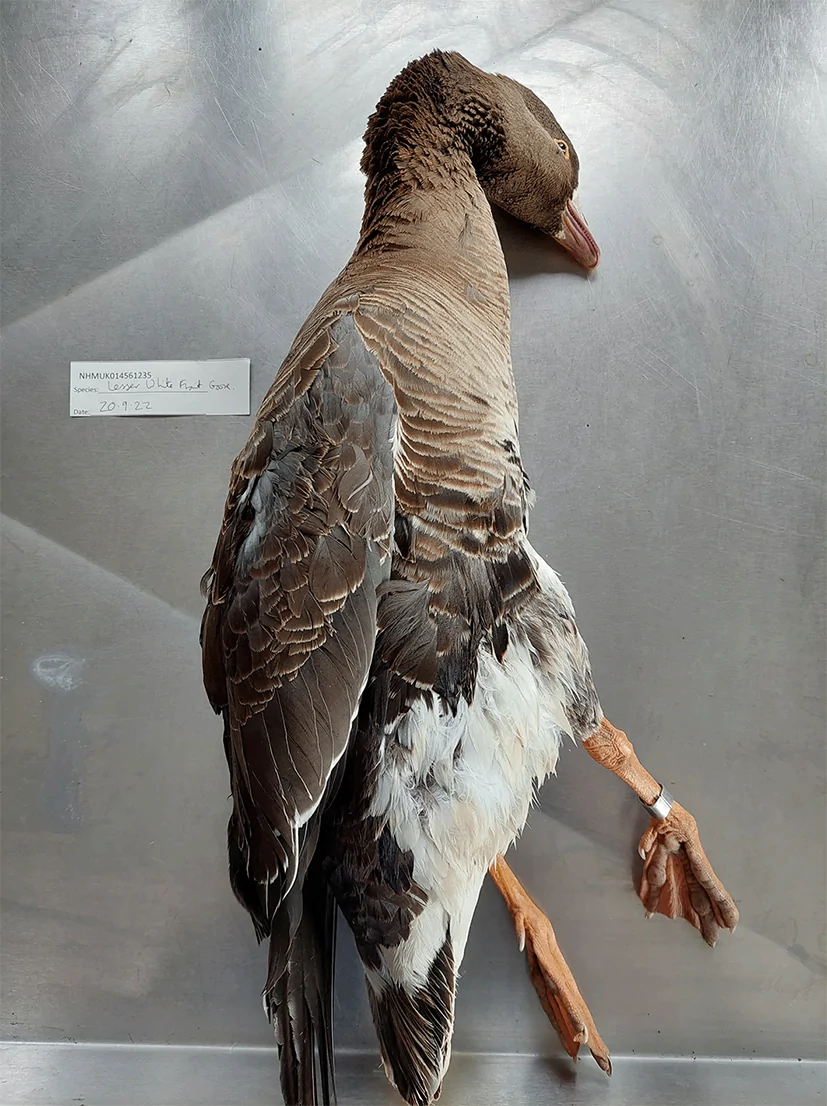
Photograph of the *Anser erythropus* (bAnsEry1) specimen used for genome sequencing.

The specimen used for genome sequencing was an adult female deceased captive specimen of *Anser erythropus* (specimen ID NHMUK014561235, ToLID bAnsEry1; Figure 1), collected from Newgounds, Gloucester, England, UK (latitude 51.73, longitude −2.40) on 2022-09-20. The specimen was collected and identified by Michelle O’Brien (Wildfowl & Wetlands Trust). Several small samples of pectoral muscle were taken and stored at −80 °C. The same specimen was used for RNA sequencing.

### Nucleic acid extraction

Detailed protocols for nucleic acid extraction developed at the Wellcome Sanger Institute (WSI) Tree of Life Core Laboratory are available on protocols.io ( Howard *et al.*, 2025). The bAnsEry1 sample was weighed and triaged to determine the appropriate extraction protocol. For HMW DNA extraction, 26 mg of muscle tissue was used. The tissue was homogenised by cryogenic disruption using the Covaris cryoPREP® Automated Dry Pulverizer. High molecular weight (HMW) DNA was extracted in the WSI Scientific Operations core using the Automated MagAttract v2 protocol. DNA was sheared into an average fragment size of 12–20 kb following the Megaruptor®3 for LI PacBio protocol. Sheared DNA was purified by manual SPRI (solid-phase reversible immobilisation). The concentration of the sheared and purified DNA was assessed using a Nanodrop spectrophotometer and Qubit Fluorometer using the Qubit dsDNA High Sensitivity Assay kit. Fragment size distribution was evaluated by running the sample on the FemtoPulse system. For this sample, the final post-shearing DNA had a Qubit concentration of 30.8 ng/μL and a yield of 1 562.00 ng, with a fragment size of 12.4 kb.

RNA was extracted from muscle tissue of bAnsEry1 in the Tree of Life Laboratory at the WSI using the RNA Extraction: Automated MagMax™ *mir*Vana protocol. The RNA concentration was assessed using a Nanodrop spectrophotometer and a Qubit Fluorometer using the Qubit RNA Broad-Range Assay kit. Analysis of the integrity of the RNA was done using the Agilent RNA 6000 Pico Kit and Eukaryotic Total RNA assay.

### PacBio HiFi library preparation and sequencing

Library preparation and sequencing were performed at the WSI Scientific Operations core. Libraries were prepared using the SMRTbell Prep Kit 3.0 (Pacific Biosciences, California, USA), following the manufacturer’s instructions. The kit includes reagents for end repair/A-tailing, adapter ligation, post-ligation SMRTbell bead clean-up, and nuclease treatment. Size selection and clean-up were performed using diluted AMPure PB beads (Pacific Biosciences). DNA concentration was quantified using a Qubit Fluorometer v4.0 (ThermoFisher Scientific) and the Qubit 1X dsDNA HS assay kit. Final library fragment size was assessed with the Agilent Femto Pulse Automated Pulsed Field CE Instrument (Agilent Technologies) using the gDNA 55 kb BAC analysis kit.

The sample was sequenced on a Revio instrument (Pacific Biosciences). The prepared library was normalised to 2 nM, and 15 μL was used for making complexes. Primers were annealed and polymerases bound to generate circularised complexes, following the manufacturer’s instructions. Complexes were purified using 1.2X SMRTbell beads, then diluted to the Revio loading concentration (200–300 pM) and spiked with a Revio sequencing internal control. The sample was sequenced on a Revio 25 M SMRT cell. The SMRT Link software (Pacific Biosciences), a web-based workflow manager, was used to configure and monitor the run and to carry out primary and secondary data analysis.

### Hi-C

***Sample preparation and crosslinking***

The Hi-C sample was prepared from 20–50 mg of frozen muscle tissue from the bAnsEry1 sample using the Arima-HiC v2 kit (Arima Genomics). Following the manufacturer’s instructions, tissue was fixed and DNA crosslinked using TC buffer to a final formaldehyde concentration of 2%. The tissue was homogenised using the Diagnocine Power Masher-II. Crosslinked DNA was digested with a restriction enzyme master mix, biotinylated, and ligated. Clean-up was performed with SPRISelect beads before library preparation. DNA concentration was measured with the Qubit Fluorometer (Thermo Fisher Scientific) and Qubit HS Assay Kit. The biotinylation percentage was estimated using the Arima-HiC v2 QC beads.

***Hi-C library preparation and sequencing***

Biotinylated DNA constructs were fragmented using a Covaris E220 sonicator and size selected to 400–600 bp using SPRISelect beads. DNA was enriched with Arima-HiC v2 kit Enrichment beads. End repair, A-tailing, and adapter ligation were carried out with the NEBNext Ultra II DNA Library Prep Kit (New England Biolabs), following a modified protocol where library preparation occurs while DNA remains bound to the Enrichment beads. Library amplification was performed using KAPA HiFi HotStart mix and a custom Unique Dual Index (UDI) barcode set (Integrated DNA Technologies). Depending on sample concentration and biotinylation percentage determined at the crosslinking stage, libraries were amplified with 10–16 PCR cycles. Post-PCR clean-up was performed with SPRISelect beads. Libraries were quantified using the AccuClear Ultra High Sensitivity dsDNA Standards Assay Kit (Biotium) and a FLUOstar Omega plate reader (BMG Labtech).

Prior to sequencing, libraries were normalised to 10 ng/μL. Normalised libraries were quantified again to create equimolar and/or weighted 2.8 nM pools. Pool concentrations were checked using the Agilent 4200 TapeStation (Agilent) with High Sensitivity D500 reagents before sequencing. Sequencing was performed using paired-end 150 bp reads on the Illumina NovaSeq X.

### RNA library preparation and sequencing

Libraries were prepared using the NEBNext® Ultra™ II Directional RNA Library Prep Kit for Illumina (New England Biolabs), following the manufacturer’s instructions. Poly(A) mRNA in the total RNA solution was isolated using oligo (dT) beads, converted to cDNA, and uniquely indexed; 14 PCR cycles were performed. Libraries were size-selected to produce fragments between 100–300 bp. Libraries were quantified, normalised, pooled to a final concentration of 2.8 nM, and diluted to 150 pM for loading. Sequencing was carried out on the Illumina NovaSeq X, generating paired-end reads.

### Genome assembly

Prior to assembly of the PacBio HiFi reads, a database of *k*-mer counts ( *k* = 31) was generated from the filtered reads using FastK. GenomeScope2 ( Ranallo-Benavidez *et al.*, 2020) was used to analyse the *k*-mer frequency distributions, providing estimates of genome size, heterozygosity, and repeat content.

The HiFi reads were assembled using Hifiasm in Hi-C phasing mode ( Cheng *et al.*, 2021, 2022), producing two haplotypes. Hi-C reads ( Rao *et al.*, 2014) were mapped to the primary contigs using bwa-mem2 ( Vasimuddin *et al.*, 2019). Contigs were further scaffolded with Hi-C data in YaHS ( Zhou *et al.*, 2023), using the --break option for handling potential misassemblies. The scaffolded assemblies were evaluated using Gfastats ( Formenti *et al.*, 2022), BUSCO ( Manni *et al.*, 2021) and MerquryFK ( Rhie *et al.*, 2020).

The mitochondrial genome was assembled using MitoHiFi ( Uliano-Silva *et al.*, 2023).

### Assembly curation

The assembly was decontaminated using the Assembly Screen for Cobionts and Contaminants ( ASCC) pipeline. TreeVal was used to generate the flat files and maps for use in curation. Manual curation was conducted primarily in PretextView and HiGlass ( Kerpedjiev *et al.*, 2018). Scaffolds were visually inspected and corrected as described by Howe *et al*. (2021). Manual corrections included 22 breaks and 125 joins. This reduced the scaffold count by 5.1%, increased the scaffold N50 by 19.8%, and increased the total assembly length by 0.6%. The curation process is described at https://gitlab.com/wtsi-grit/rapid-curation . PretextSnapshot was used to generate a Hi-C contact map of the final assembly.

### Assembly quality assessment

The MerquryFK tool ( Rhie *et al.*, 2020) was run in a Singularity container ( Kurtzer *et al.*, 2017) to evaluate *k*-mer completeness and assembly quality for both haplotypes using the *k*-mer database ( *k* = 31) computed prior to genome assembly. The analysis outputs included assembly QV scores and completeness statistics.

The genome was analysed using the BlobToolKit pipeline, a Nextflow implementation of the earlier Snakemake version ( Challis *et al.*, 2020). The pipeline aligns PacBio reads using minimap2 ( Li, 2018) and SAMtools ( Danecek *et al.*, 2021) to generate coverage tracks. It runs BUSCO ( Manni *et al.*, 2021) using lineages identified from the NCBI Taxonomy ( Schoch *et al.*, 2020). For the three domain-level lineages, BUSCO genes are aligned to the UniProt Reference Proteomes database ( Bateman *et al.*, 2023) using DIAMOND blastp ( Buchfink *et al.*, 2021). The genome is divided into chunks based on the density of BUSCO genes from the closest taxonomic lineage, and each chunk is aligned to the UniProt Reference Proteomes database with DIAMOND blastx. Sequences without hits are chunked using seqtk and aligned to the NT database with blastn ( Altschul *et al.*, 1990). The BlobToolKit suite consolidates all outputs into a blobdir for visualisation. The BlobToolKit pipeline was developed using nf-core tooling ( Ewels *et al.*, 2020) and MultiQC ( Ewels *et al.*, 2016), with containerisation through Docker ( Merkel, 2014) and Singularity ( Kurtzer *et al.*, 2017).

## Genome sequence report

### Sequence data

**Figure 2.**
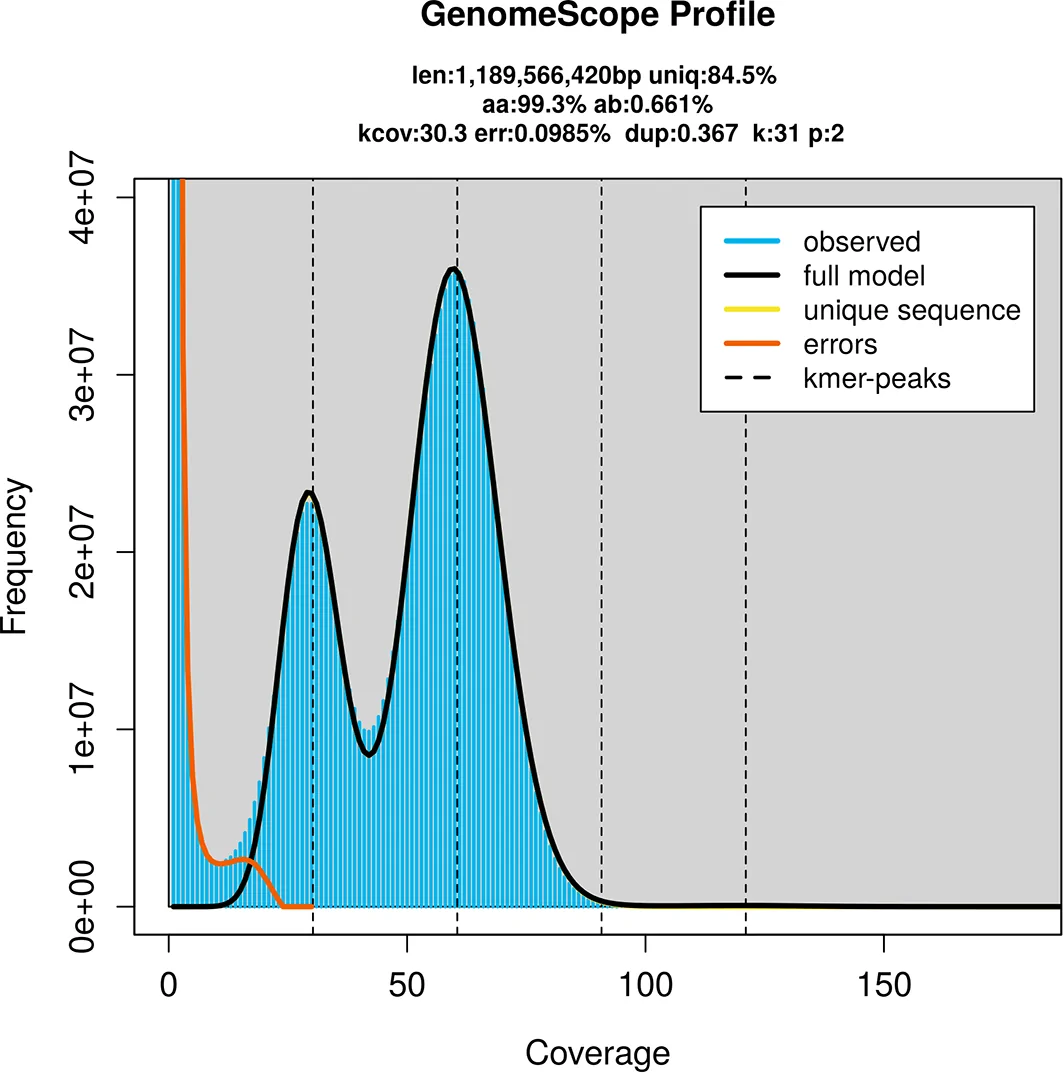
Frequency distribution of *k*-mers generated using GenomeScope2. The plot shows observed and modelled *k*-mer spectra, providing estimates of genome size, heterozygosity, and repeat content based on unassembled sequencing reads.

PacBio sequencing of the *Anser erythropus* specimen generated 74.47 Gb (gigabases) from 8.10 million reads, which were used to assemble the genome. GenomeScope2.0 analysis estimated the haploid genome size at 1 190.10 Mb, with a heterozygosity of 0.66% and repeat content of 15.53% ( Figure 2). These estimates guided expectations for the assembly. Based on the estimated genome size, the sequencing data provided approximately 61× coverage. Hi-C sequencing produced 72.35 Gb from 239.57 million reads, which were used to scaffold the assembly. RNA sequencing data were also generated and are available in public sequence repositories. Table 1 summarises the specimen and sequencing details.

**Table 1.** Specimen and sequencing data for *Anser erythropus* (BioProject PRJEB74599).

Platform	PacBio HiFi	Hi-C	RNA-seq
**ToLID**	bAnsEry1	bAnsEry1	bAnsEry1
**Specimen ID**	NHMUK014561235	NHMUK014561235	NHMUK014561235
**BioSample (source individual)**	SAMEA113398961	SAMEA113398961	SAMEA113398961
**BioSample (tissue)**	SAMEA114299713	SAMEA114299713	SAMEA114299713
**Tissue**	muscle	muscle	muscle
**Instrument**	Revio	Illumina NovaSeq X	Illumina NovaSeq X
**Run accessions**	ERR12875151	ERR12861080	ERR13493937
**Read count total**	8.10 million	239.57 million	25.49 million
**Base count total**	74.47 Gb	72.35 Gb	7.70 Gb

### Assembly statistics

The genome was assembled into two haplotypes using Hi-C phasing. Haplotype 1 was curated to chromosome level, while haplotype 2 was assembled to scaffold level. The final assembly has a total length of 1 339.88 Mb in 925 scaffolds, with 920 gaps, and a scaffold N50 of 80.25 Mb ( Table 2).

**Table 2.** Genome assembly statistics for *Anser erythropus.*

Genome assembly	Haplotype 1	Haplotype 2
**Assembly name**	bAnsEry1.hap1.1	bAnsEry1.hap2.1
**Assembly accession**	GCA_965277985.1	GCA_965277975.1
**Assembly level**	chromosome	scaffold
**Span (Mb)**	1 339.88	1 168.02
**Number of chromosomes**	41	scaffold-level
**Number of contigs**	1 845	1 247
**Contig N50**	2.38 Mb	2.75 Mb
**Number of scaffolds**	925	524
**Scaffold N50**	80.25 Mb	70.87 Mb
**Longest scaffold length (Mb)**	218.07	216.84
**Sex chromosomes**	W and Z	-
**Organelles**	Mitochondrion: 16.74 kb	-

**Figure 3.**
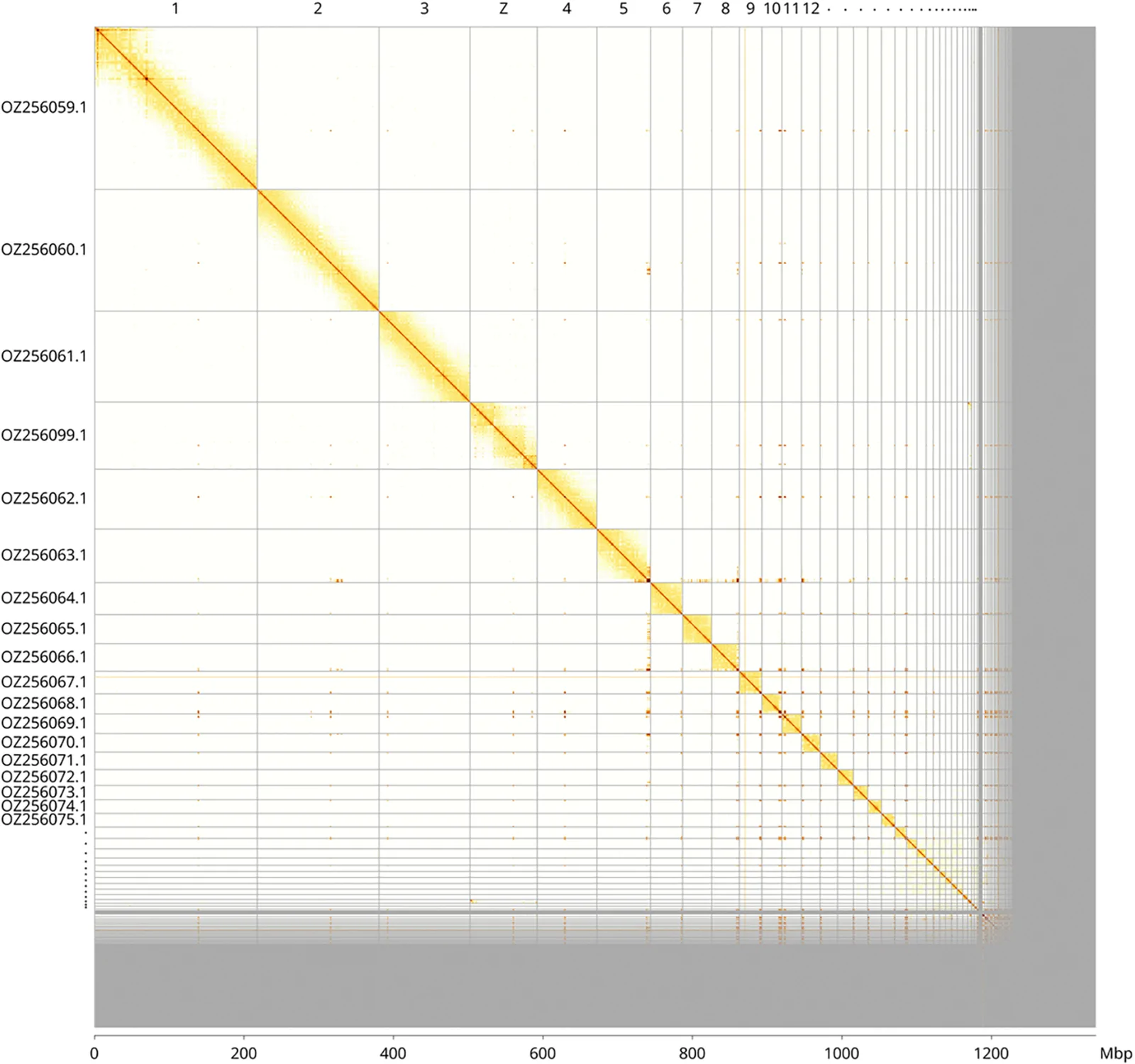
Hi-C contact map of the *Anser erythropus* genome assembly. Assembled chromosomes are shown in order of size and labelled along the axes, with a megabase scale shown below. The plot was generated using PretextSnapshot.

Most of the haplotype 1 assembly sequence (88.67%) was assigned to 41 chromosomal-level scaffolds, representing 39 autosomes and the W and Z sex chromosomes. These chromosome-level scaffolds, confirmed by Hi-C data, are named according to size ( Figure 3; Table 3).

**Table 3.** Chromosomal pseudomolecules in the haplotype 1 genome assembly of *Anser erythropus* bAnsEry1.

INSDC accession	Molecule	Length (Mb)	GC%
OZ256059.1	1	218.07	40.50
OZ256060.1	2	162.73	40
OZ256061.1	3	122.06	40.50
OZ256062.1	4	80.25	40.50
OZ256063.1	5	71.47	42
OZ256064.1	6	43.08	42
OZ256065.1	7	39.01	42.50
OZ256066.1	8	36.65	43.50
OZ256067.1	9	30.30	44.50
OZ256068.1	10	27.04	46
OZ256069.1	11	25.98	45.50
OZ256070.1	12	25.15	44
OZ256071.1	13	23.19	44.50
OZ256072.1	14	21.42	46
OZ256073.1	15	19.06	46
OZ256074.1	16	18.22	47.50
OZ256075.1	17	18.12	47
OZ256076.1	18	15.45	49
OZ256077.1	19	13.94	50
OZ256078.1	20	12.17	47.50
OZ256079.1	21	9.87	49
OZ256080.1	22	8.13	53
OZ256081.1	23	8	51.50
OZ256082.1	24	7.93	50
OZ256083.1	25	7.62	53
OZ256084.1	26	7.42	52.50
OZ256085.1	27	6.54	50
OZ256086.1	28	4.03	58.50
OZ256087.1	29	3.24	59
OZ256088.1	30	2.10	57.50
OZ256089.1	31	1.34	54
OZ256090.1	32	0.78	60
OZ256091.1	33	0.71	56
OZ256092.1	34	0.57	57.50
OZ256093.1	35	0.51	51
OZ256094.1	36	0.45	61.50
OZ256095.1	37	0.24	57.50
OZ256096.1	38	0.22	61.50
OZ256097.1	39	0.21	58.50
OZ256098.1	W	5.22	46.50
OZ256099.1	Z	89.52	41

The mitochondrial genome was also assembled (length 16.74 kb, OZ256100.1). This sequence is included as a contig in the multifasta file of the genome submission and as a standalone record.

### Assembly quality metrics

**Figure 4.**
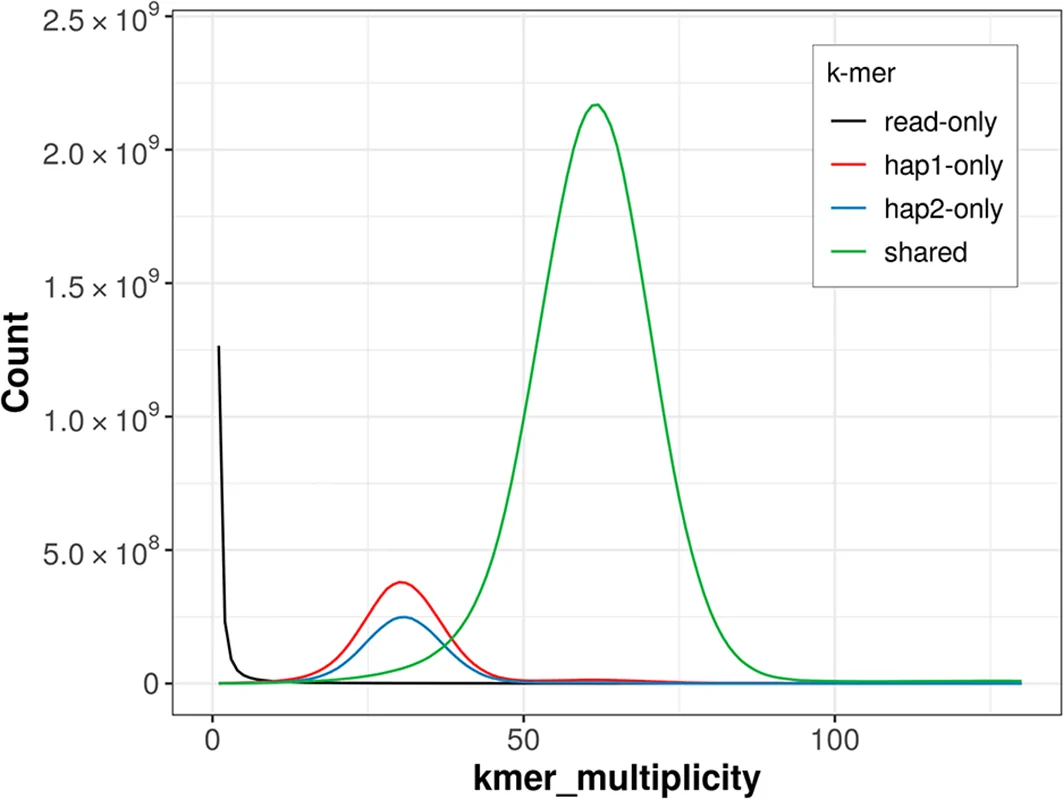
Evaluation of *k*-mer completeness using MerquryFK. This plot illustrates the recovery of *k*-mers from the original read data in the final assemblies. The horizontal axis represents *k*-mer multiplicity, and the vertical axis shows the number of *k*-mers. The black curve represents *k*-mers that appear in the reads but are not assembled. The green curve corresponds to *k*-mers shared by both haplotypes, and the red and blue curves show *k*-mers found only in one of the haplotypes.

For haplotype 1, the estimated QV is 60.7, and for haplotype 2, 61.6. When the two haplotypes are combined, the assembly achieves an estimated QV of 61.1. The *k*-mer completeness is 88.71% for haplotype 1, 82.26% for haplotype 2, and 99.69% for the combined haplotypes ( Figure 4).

**Figure 5.**
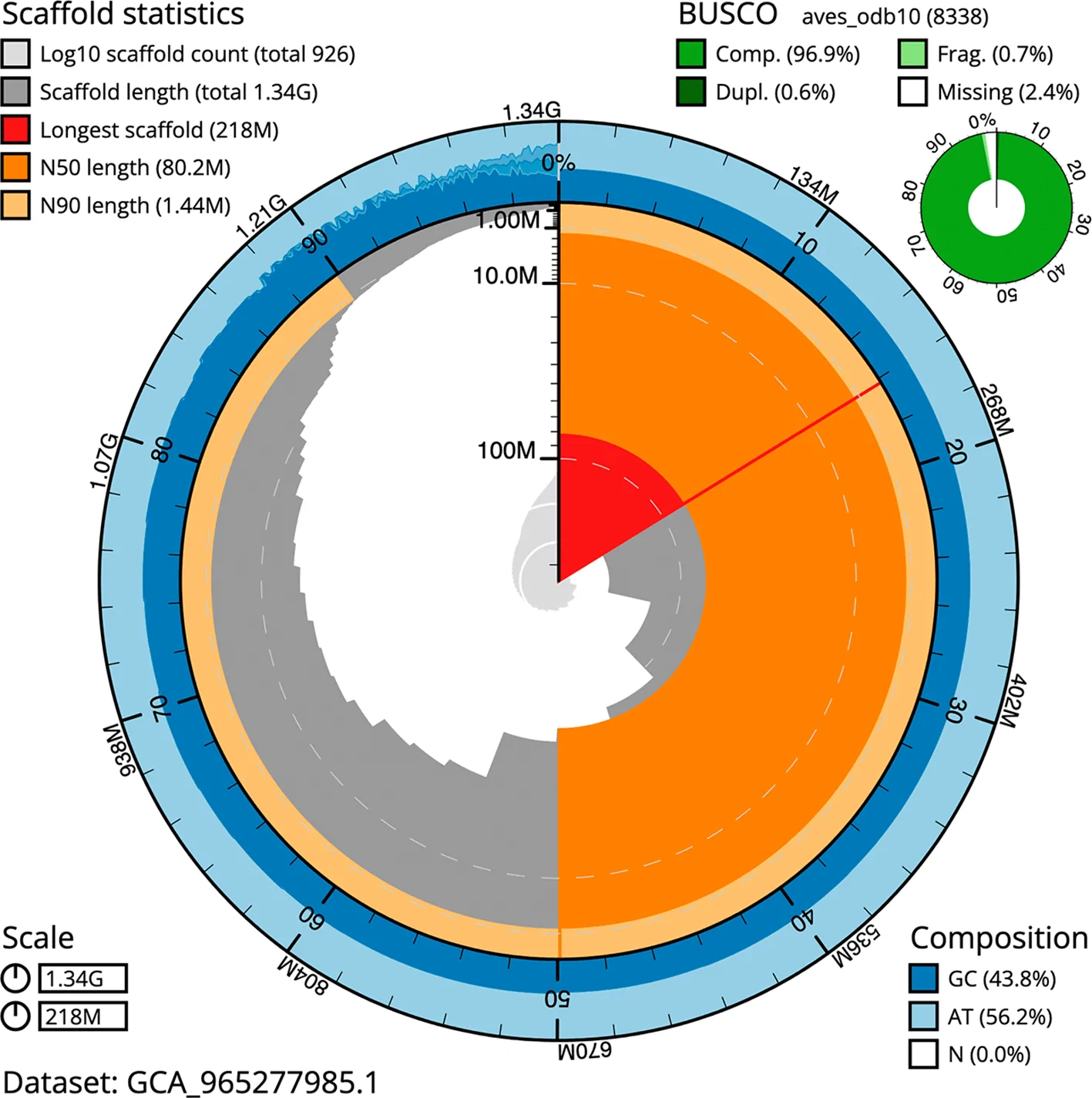
Assembly metrics for bAnsEry1.hap1.1. The BlobToolKit snail plot provides an overview of assembly metrics and BUSCO gene completeness. The circumference represents the length of the whole genome sequence, and the main plot is divided into 1 000 bins around the circumference. The outermost blue tracks display the distribution of GC, AT, and N percentages across the bins. Scaffolds are arranged clockwise from longest to shortest and are depicted in dark grey. The longest scaffold is indicated by the red arc, and the deeper orange and pale orange arcs represent the N50 and N90 lengths. A light grey spiral at the centre shows the cumulative scaffold count on a logarithmic scale. A summary of complete, fragmented, duplicated, and missing BUSCO genes in the set is presented at the top right. An interactive version of this figure can be accessed on the BlobToolKit viewer.

**Figure 6.**
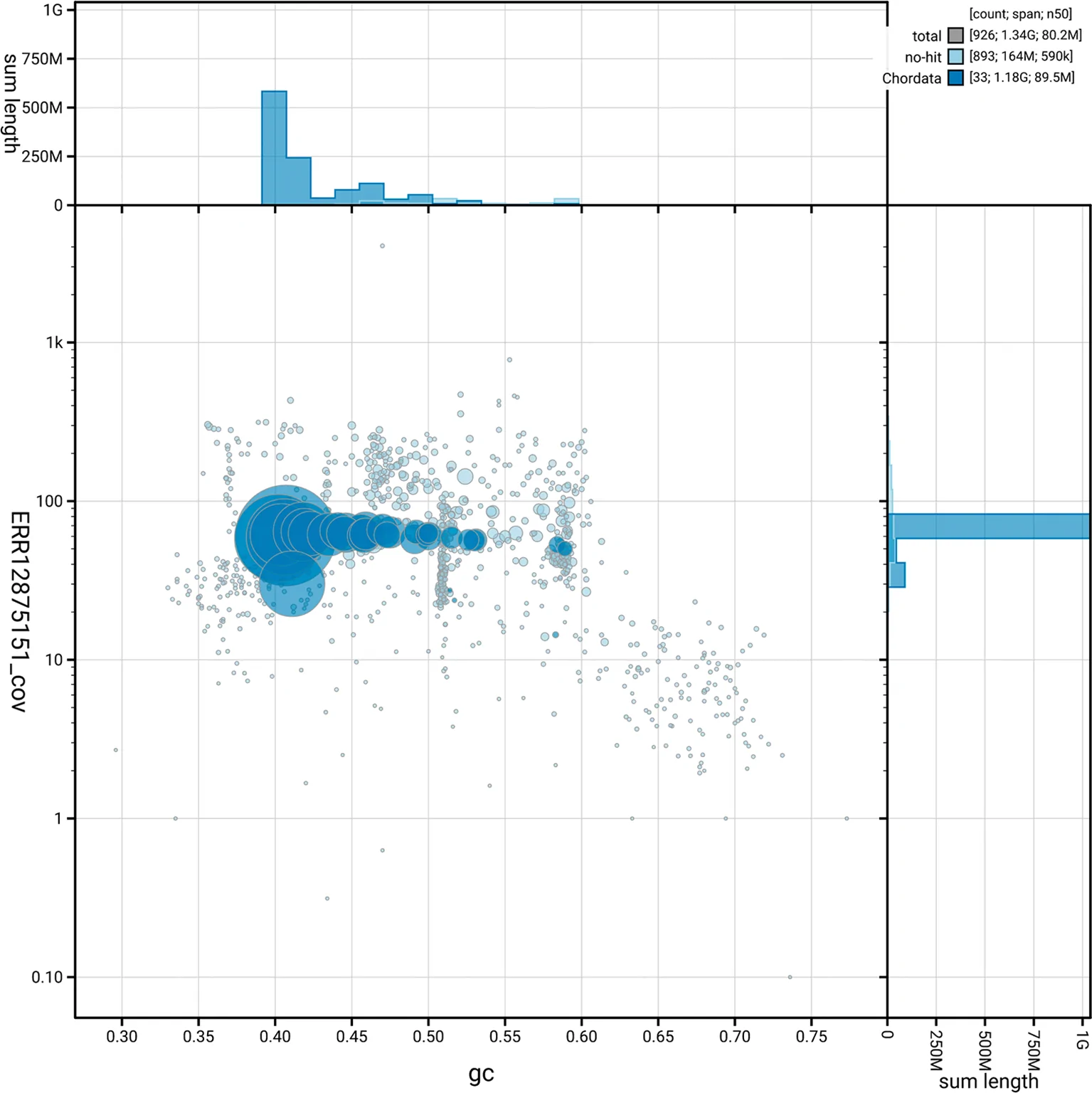
BlobToolKit blob plot for bAnsEry1.hap1.1. The plot shows base coverage (vertical axis) and GC content (horizontal axis). The circles represent scaffolds, with the size proportional to scaffold length and the colour representing phylum membership. The histograms along the axes display the total length of sequences distributed across different levels of coverage and GC content. An interactive version of this figure is available on the BlobToolKit viewer.

BUSCO analysis using the aves_odb10 reference set ( *n* = 8 338) identified 96.9% of the expected gene set (single = 96.3%, duplicated = 0.6%) in haplotype 1. For haplotype 2, BUSCO v.5.7.1 analysis identified 92.6% of the expected gene set (single = 92.2%, duplicated = 0.3%). The snail plot in Figure 5 summarises the scaffold length distribution and other assembly statistics for haplotype 1. The blob plot in Figure 6 shows the distribution of scaffolds by GC proportion and coverage for haplotype 1.

Table 4 lists the assembly metric benchmarks adapted from Rhie *et al.* (2021) and the Earth BioGenome Project Report on Assembly Standards January 2026. The EBP metric, calculated for the haplotype 1, is **6.7.Q60**.

**Table 4.** Earth Biogenome Project summary metrics for the *Anser erythropus* assembly.

Measure	Value	Benchmark
EBP summary (haplotype 1)	6.7.Q60	6.C.Q40
Contig N50 length	2.38 Mb	≥ 1 Mb
Scaffold N50 length	80.25 Mb	= chromosome N50
Consensus quality (QV)	Haplotype 1: 60.7; haplotype 2: 61.6; combined: 61.1	≥ 40
*k*-mer completeness	Haplotype 1: 88.71%; Haplotype 2: 82.26%; combined: 99.69%	≥ 95%
BUSCO	C:96.9% [S:96.3%, D:0.6%], F:0.7%, M:2.4%, n:8 338	S > 90%; D < 5%
Percentage of assembly assigned to chromosomes	88.67%	≥ 90%

**Table 5.** Software versions and sources used for *Anser erythropus.*

Software	Version	Source
BLAST	2.14.0	ftp://ftp.ncbi.nlm.nih.gov/blast/executables/blast+/
BlobToolKit	4.4.5	https://github.com/blobtoolkit/blobtoolkit
BUSCO	5.7.1	https://gitlab.com/ezlab/busco
bwa-mem2	2.2.1	https://github.com/bwa-mem2/bwa-mem2
DIAMOND	2.1.8	https://github.com/bbuchfink/diamond
fasta_windows	0.2.4	https://github.com/tolkit/fasta_windows
FastK	1.1	https://github.com/thegenemyers/FASTK
GenomeScope2.0	2.0.1	https://github.com/tbenavi1/genomescope2.0
Gfastats	1.3.6	https://github.com/vgl-hub/gfastats
Hifiasm	0.19.8-r603	https://github.com/chhylp123/hifiasm
HiGlass	1.13.4	https://github.com/higlass/higlass
MerquryFK	1.1.2	https://github.com/thegenemyers/MERQURY.FK
Minimap2	2.28-r1209	https://github.com/lh3/minimap2
MitoHiFi	3	https://github.com/marcelauliano/MitoHiFi
MultiQC	1.14; 1.17 and 1.18	https://github.com/MultiQC/MultiQC
Nextflow	24.10.4	https://github.com/nextflow-io/nextflow
PretextSnapshot	0.0.5	https://github.com/sanger-tol/PretextSnapshot
PretextView	1.0.3	https://github.com/sanger-tol/PretextView
samtools	1.21	https://github.com/samtools/samtools
sanger-tol/ascc	0.1.0	https://github.com/sanger-tol/ascc
sanger-tol/blobtoolkit	v0.7.1	https://github.com/sanger-tol/blobtoolkit
sanger-tol/curationpretext	1.4.2	https://github.com/sanger-tol/curationpretext
Seqtk	1.3	https://github.com/lh3/seqtk
Singularity	3.9.0	https://github.com/sylabs/singularity
TreeVal	1.4.0	https://github.com/sanger-tol/treeval
YaHS	1.2a.2	https://github.com/c-zhou/yahs

**Notes:** The EBP summary uses log10(Contig N50); chromosome-level (C) or log10(Scaffold N50); Q (Merqury QV). BUSCO: C = complete; S = single-copy; D = duplicated; F = fragmented; M = missing; n = orthologues.

## Author information

Contributors are listed at the following links:

• Members of the Natural History Museum Genome Acquisition Lab
• Members of the Wellcome Sanger Institute Tree of Life Management, Samples and Laboratory team
• Members of Wellcome Sanger Institute Scientific Operations – Sequencing Operations
• Members of the Wellcome Sanger Institute Tree of Life Core Informatics team
• Members of the Tree of Life Core Informatics collective
• Members of the Darwin Tree of Life Consortium

## Wellcome Sanger Institute – Legal and Governance

The materials that have contributed to this genome note have been supplied by a Darwin Tree of Life Partner. The submission of materials by a Darwin Tree of Life Partner is subject to the **‘Darwin Tree of Life Project Sampling Code of Practice’**, which can be found in full on the Darwin Tree of Life website. By agreeing with and signing up to the Sampling Code of Practice, the Darwin Tree of Life Partner agrees they will meet the legal and ethical requirements and standards set out within this document in respect of all samples acquired for, and supplied to, the Darwin Tree of Life Project. Further, the Wellcome Sanger Institute employs a process whereby due diligence is carried out proportionate to the nature of the materials themselves, and the circumstances under which they have been/are to be collected and provided for use. The purpose of this is to address and mitigate any potential legal and/or ethical implications of receipt and use of the materials as part of the research project, and to ensure that in doing so we align with best practice wherever possible. The overarching areas of consideration are:

• Ethical review of provenance and sourcing of the material
• Legality of collection, transfer and use (national and international)

Each transfer of samples is further undertaken according to a Research Collaboration Agreement or Material Transfer Agreement entered into by the Darwin Tree of Life Partner, Genome Research Limited (operating as the Wellcome Sanger Institute), and in some circumstances, other Darwin Tree of Life collaborators.

## Data Availability

European Nucleotide Archive: Anser erythropus (lesser white-fronted goose). Accession number PRJEB74599. The genome sequence is released openly for reuse. The *Anser erythropus* genome sequencing initiative is part of the Darwin Tree of Life Project (PRJEB40665), the Sanger Institute Tree of Life Programme (PRJEB43745) and the Vertebrate Genomes Project (PRJNA489243). All raw sequence data and the assembly have been deposited in INSDC databases. The genome will be annotated using available RNA-Seq data and presented through the Ensembl pipeline at the European Bioinformatics Institute. Raw data and assembly accession identifiers are reported in Tables 1 and 2. Production code used in genome assembly at the WSI Tree of Life is available at https://github.com/sanger-tol . Table 5 lists software versions used in this study.
