# Supplementary material for: The genome sequence of the lesser white-fronted goose, Anser erythropus (Linnaeus, 1758) (Anseriformes: Anatidae)
Source: Wellcome Open Res. 2026 Jun 13;11:364. [Version 1] doi: 10.12688/wellcomeopenres.26718.1 (PMC13555084; doi:10.12688/wellcomeopenres.26718.1)
Supplement: Peer review history

# Reviewer report: Reviewer response for version 1

**DOI:** 10.21956/wellcomeopenres.29420.r160683

**Published:** 2026-07-24

**Authors:** Jerome Fuchs

**Affiliations:** 1 Muséum national d'Histoire naturelle, Paris, France

The paper provides a chromosome level assembly for a Vulnerable species, the lesser white-fronted gosse. Amount of data and methodology are highly appropriate. 

 i have a couple of minor questions:

 - the authors mention a NHMUK ID number. Does this refer to the tissue sample ? a preserved study skin or skeleton ?

 - maybe it worth mentioning that the number of chromosome estimated here (2n=80) slighlty differs with what is know with Anser karyotype data (https://sites.unipampa.edu.br/birdchromosomedatabase/chromosome-number-in-birds/) but is in line with genome sequencing projects.

Are sufficient details of methods and materials provided to allow replication by others?

Yes

Is the rationale for creating the dataset(s) clearly described?

Yes

Are the datasets clearly presented in a useable and accessible format?

Yes

Are the protocols appropriate and is the work technically sound?

Yes

Reviewer Expertise:

Birds evolution

I confirm that I have read this submission and believe that I have an appropriate level of expertise to confirm that it is of an acceptable scientific standard.

# Reviewer report: Reviewer response for version 1

**DOI:** 10.21956/wellcomeopenres.29420.r158565

**Published:** 2026-07-02

**Authors:** Andrew Sweet

**Affiliations:** 1 Arkansas State University, Jonesboro, Arkansas, USA

The authors sequenced and assembled the nuclear and mitochondrial genomes from a Lesser White-fronted Goose. The resulting genome included chromosome-level information, and multiple haplotype assemblies. This genome is an important resource for conservation and biodiversity genomics.

 I have a few questions/comments that I think will improve the manuscript:

- The sentence about variable food sources (end of paragraph 2) is confusing without context of the wintering and breeding grounds. Please provide more details about the limited range of breeding and wintering grounds here.
- What was the purpose of the RNA sequencing data? I presumed it was to aid in annotation, but there was no annotation work in this report. Regardless, would RNA data from muscle tissue alone be sufficient for annotation? Also, what type of muscle tissue was used for RNAseq?
- Did you confirm that the mitogenome was from Lesser White-fronted Goose, and not from Greater White-fronted Goose as was shown in birds from the Swedish breeding programme?

Are sufficient details of methods and materials provided to allow replication by others?

Yes

Is the rationale for creating the dataset(s) clearly described?

Yes

Are the datasets clearly presented in a useable and accessible format?

Yes

Are the protocols appropriate and is the work technically sound?

Yes

Reviewer Expertise:

Evolutionary genomics, host-parasite evolution, avian evolution

I confirm that I have read this submission and believe that I have an appropriate level of expertise to confirm that it is of an acceptable scientific standard.
